# Supplementary material for: A quantitative approach to evaluating the GWP timescale through implicit discount rates
Source: Earth Syst Dyn. Author manuscript; Available in PMC 2019 Aug 27. (PMC6711200; doi:10.5194/esd-2018-6)
Supplement: SI [file NIHMS1043049-supplement-SI.zip › MetricsPaperCode/README for Metrics Paper Code.docx]

**README for Metrics Paper Code**

The code in this folder is designed to support the paper “A quantitative approach to evaluating the GWP timescale through implicit discount rates” by Marcus Sarofim and Mike Giordano. The paper is being prepared for submission to the journal Earth Systems Dynamics. The code was mainly developed and written by the author, Marcus Sarofim, with input from Mike Giordano, Sarah Menassian, and Jameel Alsalam. However, any errors or inelegant coding are the responsibility of the author.

The United States Environmental Protection Agency (EPA) GitHub project code is provided on an "as is" basis and the user assumes responsibility for its use.  EPA has relinquished control of the information and no longer has responsibility to protect the integrity , confidentiality, or availability of the information.  Any reference to specific commercial products, processes, or services by service mark, trademark, manufacturer, or otherwise, does not constitute or imply their endorsement, recommendation or favoring by EPA.  The EPA seal and logo shall not be used in any manner to imply endorsement of any commercial product or activity by EPA or the United States Government.

This code is considered public domain under the Creative Commons CC0 1.0 Universal (CC0 1.0) Public Domain Dedication

Folders: Main level, data, results, src.

data:

The data folder includes 4 files. 1 file holds exogenous projections of GDP (from the Nordhaus DICE 2016 model at http://www.econ.yale.edu/~nordhaus/homepage/DICEmodels09302016.htm) and the other 4 files contain GHG concentration and forcing data from the 4 RCPs (from <http://www.pik-potsdam.de/~mmalte/rcps/>).

The source folder contains one file, metricsfunctions.R, which contains all the functions called by the programs in the main folder.

The results folder holds any output generated by the code.

And finally, the main folder contains the R project and 4 different pieces of R code, each of which is designed to produce a part of the analysis used in the paper.

src/metricsfunctions.R:

This code is divided into several clusters of functions.

The first set of functions addresses concentrations and forcing calculations from CO2, methane, N2O, and any fluorinated compound (HFCs, PFCs, SF6).

The next cluster of functions returns a net present value of vector given a discount rate. Trapsum2 is the fastest of the functions, which is important when running the full sensitivity (a 3 minute calculation instead of an hour long calculation).

The next function looks at temperature change in a given year given a pulse in year zero, a background forcing, a forcing imbalance at year zero, and a climate sensitivity. This uses the temperature functions from the IPCC AR5 chapter 8 supplementary material (8.SM.11.2).

The next set of functions can calculate a traditional GWP (with constant concentrations) based on a vector of forcings and a timescale of integration.

Finally, the “calcGWProot” functions calculate an optimal GWP timescale given a ratio of impact between the gas and CO2 (again, assuming constant concentrations). Non-monotonic damage functions (like for N2O) will cause problems in solving.

Main level:

FullSensitivityESD.R:

This code calculates the ratio of CH4 impacts to CO2 impacts over a range of parameters and discount rates, along with the GWP equivalent timescales that would match that damage ratio.

Expects concentration data and GDP data as an input.

Produces:

Figure 2 (has been replotted in other software for the final paper version), but fig2dummy.pdf is the same data.

doubleaxis.pdf: the same as Figure 2, but with the first axis as the CH4/CO2 ratio, and the 2^nd^ axis as the equivalent GWP length.

values used for Table 1

finalframeESD.csv: first six columns represent the different sensitivity assumptions, and then the following columns represent the ratio of net present discounted impacts of CH4 over CO2 at all the different discount rates examined.

discountquantESD.csv: contains the min, max, 90/10, 75/25, and median values of the above ratio for any given discount rate.

GWPquantESD.csv: GWP equivalent timescales are calculated for each of the discountquant values.

centralparameters.R:

This code does the same calculations as the FullSensitivityESD code, except only for the central value of each parameter. But where FullSensitivity only keeps the ratios of the net present discounted impacts, this code keeps all the intermediate calculations by year and gas: radiative forcing, temperature, raw damages, and discounted damages.

This code also expects concentration and GDP projections.

The code produces:

Figure 1 (Figure1.gridplot.pdf)

calculates values for Table 2 (CH4 only)

Figure SI.3 (CH4 only) (CH4damageratio.pdf)

nonCO2ESD.R:

As for above code. but for HFC and N2O.

Produces:

data for Table 2 (HFC, N2O)

Figure SI.1 (n2ogridplot.pdf)

Figure SI.3 (N2O) (N2Odamageratio.pdf)

GWPtimescalecalc.R:

This code calculates the standard IPCC GWP (e.g., ratio of integrated forcing based on IPCC equations assuming constant concentrations) for a range of timeframes.

Takes RCP8.5 concentrations as an input (for first year concentrations).

Produces:

Figure SI.2 (N2OGWP.pdf and CH4GWP.pdf)

results:

All the codes write to this folder. Possible outputs:

discountquantESD.csv: produced by FullSensitivityESD.R

finalframeESD.csv: produced by FullSensitivityESD.R

GWPquantESD.csv: produced by FullSensitivityESD.R

Figure SI.2 (N2OGWP.pdf and CH4GWP.pdf)

Figure SI.1 (n2ogridplot.pdf)

Figure SI.3 (N2O) (N2Odamageratio.pdf)

Figure 1 (Figure1.gridplot.pdf)

Figure SI.3 (CH4 only) (CH4damageratio.pdf)

alternate figures:

fig2dummy.pdf: produced by FullsensitivityESD.R. Not as pretty as the real figure 2, but the same data.

doubleaxis.pdf: the same as Figure 2, but with the first axis as the CH4/CO2 ratio, and the 2^nd^ axis as the equivalent GWP length.
