# Supplementary material for: A quantitative approach to evaluating the GWP timescale through implicit discount rates
Source: Earth Syst Dyn. Author manuscript; Available in PMC 2019 Aug 27. (PMC6711200; doi:10.5194/esd-2018-6)
Supplement: SI [file NIHMS1043049-supplement-SI.zip › esd-9-1013-2018-supplement-title-page.pdf]

Supplement of Earth Syst. Dynam., 9, 1013–1024, 2018
https://doi.org/10.5194/esd-9-1013-2018-supplement
© Author(s) 2018. This work is distributed under
the Creative Commons Attribution 4.0 License.

Earth System Dynamics
Open Access

Supplement of

A quantitative approach to evaluating the GWP timescale through implicit discount rates

Marcus C. Sarofim and Michael R. Giordano

Correspondence to: Marcus C. Sarofim (sarofim.marcus@epa.gov)

- esd-9-1013-2018-supplement-title-page.pdf
- MetricsPaperCode
  - .Rhistory
  - .Rproj.user
    - D0FDAD79
    - shared
  - CentralParameters.R
  - FullSensitivityESD.R
  - GWPtimescalecalc.R
  - MetricsSensitivityProject.Rproj
  - README for Metrics Paper Code.docx
  - data
    - gdpovertime.csv
    - rcp3PDgasconcs.csv
    - rcp45gasconcs.csv
    - rcp6gasconcs.csv
    - rcp85gasconcs.csv
  - nonCO2ESD.R
  - results
    - CH4GWP.pdf
    - CH4damageratio.pdf
    - Figure1.gridplot.pdf
    - GWPquantESD.csv
    - N2OGWP.pdf
    - N2Odamageratio.pdf
    - discountquantESD.csv

    * doubleaxis.pdf
    * fig2dummy.pdf
    * n2ogridplot.pdf
  - src
    * metricsfunctions.R
- Sarofim_Metrics_ESD_SI_6_1_18_clean.pdf

The copyright of individual parts of the supplement might differ from the CC BY 4.0 License.
