# Supplementary material for: A quantitative approach to evaluating the GWP timescale through implicit discount rates
Source: Earth Syst Dyn. Author manuscript; Available in PMC 2019 Aug 27. (PMC6711200; doi:10.5194/esd-2018-6)
Supplement: SI [file NIHMS1043049-supplement-SI.zip › Sarofim_Metrics_ESD_SI_6_1_18_clean.pdf]

S1. Expanded discussion of non-methane gases.

The main text includes a short discussion on gases other than CH_4 and CO_2, which is expanded here. In particular, as the 3rd most important well-mixed GHG (by standard metrics), we show an analysis of N_2O impacts. However, similar results would be found for any GHG with a century-scale lifetime, just as similar results to the CH_4 analysis apply for any GHG with a decadal lifetime.

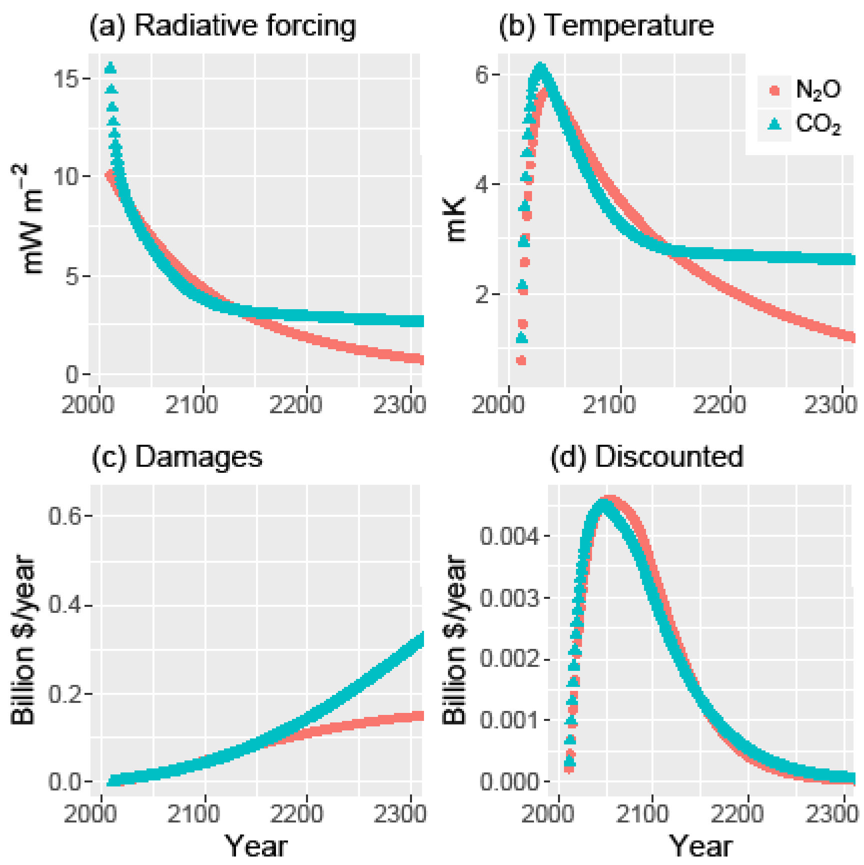

Figure S1: The impact of an emission pulse of N_2O compared to an emission pulse of 312 times as much CO_2 (the CO_2 quantity being chosen to make the integrated damages at a 3% discount rate equivalent). Radiative forcing (a), Temperature(b), damages (c), and discounted damages (3%, d). The underlying scenario is RCP6.0, with other parameters at their central values.

Figure S1 is the equivalent figure to Fig. 1 of the main text, but for N_2O. For all 4 outcomes (radiative forcing, temperature, damages, and discounted damages) the impact of an emission pulse of CO_2 is similar to the impact of an emission pulse of N_2O. After that time, the long lifetime of CO_2 causes the CO_2 function to diverge from the N_2O functions, except in the case

where discounting erases the damages. The similarity of these functions means that the relative radiative forcing and damages of N_2O to CO_2 are less sensitive to timescale and discount rate than for shorter lived gases.

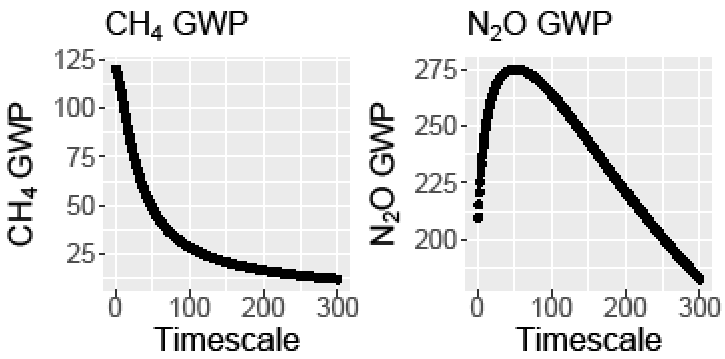

Figure S2: GWP as a function of time horizon for N_2O and CH_4.

An examination of the GWP as a function of timescale demonstrates the difference in the sensitivity of the N_2O GWP to timescale compared to sensitivity of the CH_4 GWP (see Fig. 2). As can be seen in the figure, the 1 year GWP is basically the ratio of instantaneous radiative forcing per ton of the emitted gas relative to CO_2. For methane, as the timescale increases, the GWP decreases monotonically due to the short lifetime. The N_2O function, however, is not monotonic. This is a result of the lifetime of CO_2 being determined by the sum of 4 exponentials, where two of those exponentials, accounting for more than 50% of the CO_2 emitted, have lifetimes substantially shorter than the N_2O lifetime, and the other 2 lifetimes being substantially longer. Therefore, as seen in Fig. S1(a), the radiative forcing of CO_2 decreases more quickly than that of N_2O for several decades, but then the rate of decrease in the radiative forcing of CO_2 slows as the short-lifetime pool of CO_2 is depleted. Because the GWP of N_2O never exceeds 275, it is not possible for a GWP timescale to be chosen for N_2O that can emulate a damage ratio of greater than 275 (as is the case at a discount rate of 3%, when the damage ratio is 312).

While the fact that one graph is monotonic and the other is not is the most striking difference between the CH_4 GWP graph and the N_2O GWP graph, there is another important difference which is the total variability. For CH_4, the difference between the instantaneous timescale and the 200-year timescale is a factor of 7. For N_2O, the difference between the peak GWP of 275 at a 52-year timescale, and either the instantaneous or the 200-year timescale, is less than 35%. That means that getting the timescale wrong for long-lived gases has a limited effect, whereas getting the timescale wrong for methane has substantial implications for the implied relative damages.

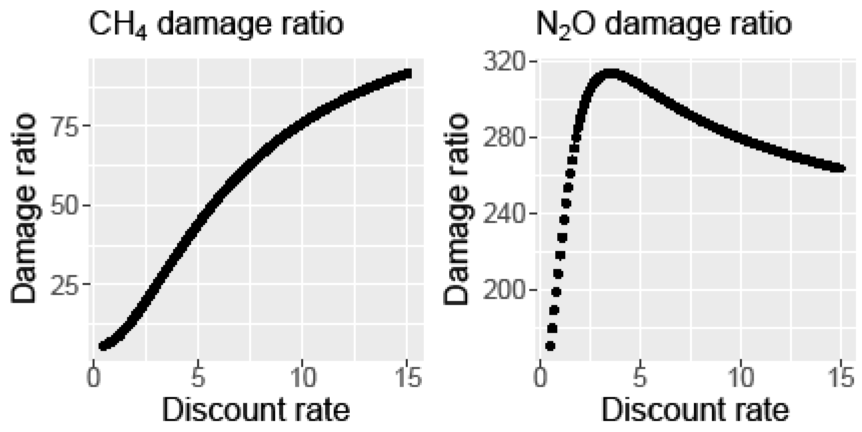

Figure S3: Using the central set of parameters, the ratio of the integrated discounted N_2O (or CH_4) damages to CO_2 damages is calculated at each discount rate.

The relative damages for N_2O and CH_4 are shown in Fig. S3. Because of the non-monotonicity of the GWP graph for N_2O, showing a damage ratio graph is more straightforward than showing an implied timescale graph. Like the N_2O GWP graph, the damage ratio graph is also non-monotonic. Where it exceeds 275, there is no exact equivalent GWP timescale. At damage ratios below 275, there are two potential equivalent timescales, one less than 52 years and one greater than 52 years: alternatively, for an equivalent timescale, there are two potential matching discount rates. For example, for a GWP timescale of 100, the ratio of N_2O damages to CO_2 damages is 264, and discount rates of 1.6% and 14.9% both produce a damage ratio of 264.

The CH_4 damage ratio graph is also shown here. When convoluted with the GWP timescale graph, it produces the median line from Fig. 2 in the main text (see Fig. S4). We see that as the discount rate approaches infinity, the damage ratio approaches the instantaneous GWP. Unlike the N_2O damage graph, the CH_4 damage graph is monotonic with discount rate.

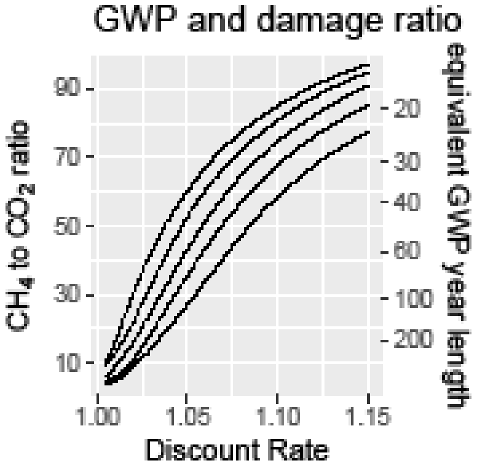

Figure S4: This shows the same data as in Fig. 2 in the main text. However, this figure is keyed to the damage ratio on the left axis, and the implied GWP timescale is shown on the right axis. Interdecile, interquartile, and median estimates are shown here as based on the sensitivity analysis.

This analysis suggests that the use of the implied timescale from the CH_4 results for all gases is a reasonable choice. First, because CH_4 is the most important non-CO_2 gas, but also because the implied timescale and damage ratios derived from the relative impacts of short-lived gases are much more sensitive to discount rates than the implied timescales and damage ratios for longer-lived gases.

S2: GDP Growth Rates

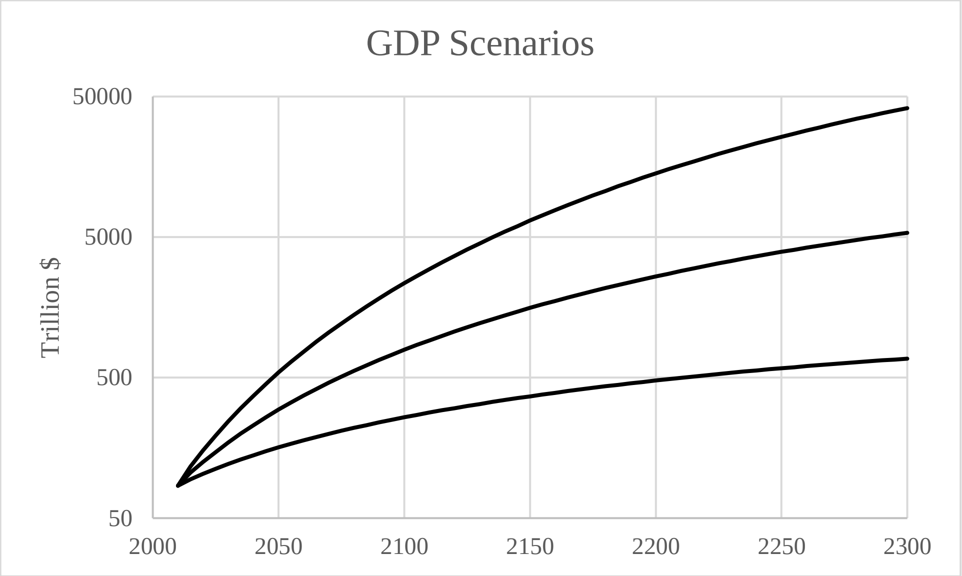

Figure S5: GDP from 2010 to 2300 for the 3 scenarios considered are displayed in this figure on a log scale.

5
