# Supplementary figures and images for: A quantitative approach to evaluating the GWP timescale through implicit discount rates
Source: Earth Syst Dyn. Author manuscript; Available in PMC 2019 Aug 27. (PMC6711200; doi:10.5194/esd-2018-6)

# N<sub>2</sub>O GWP

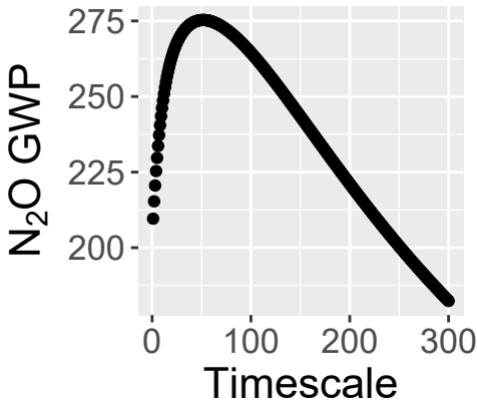

Supplement: SI [file NIHMS1043049-supplement-SI.zip › MetricsPaperCode/results/N2OGWP.pdf]

# $\text{N}_2\text{O}$ damage ratio

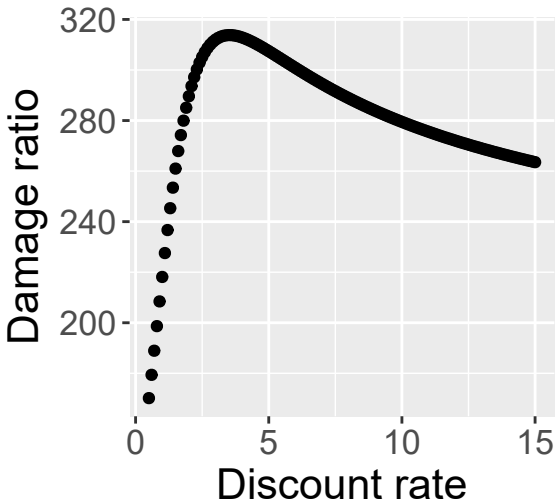

Supplement: SI [file NIHMS1043049-supplement-SI.zip › MetricsPaperCode/results/N2Odamageratio.pdf]

(a) Radiative forcing

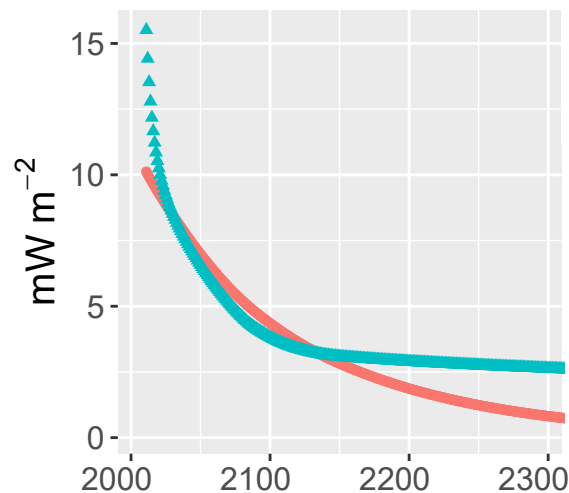

(b) Temperature

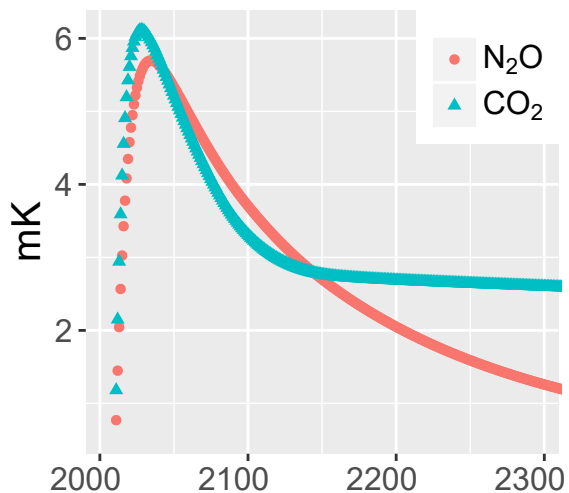

(c) Damages

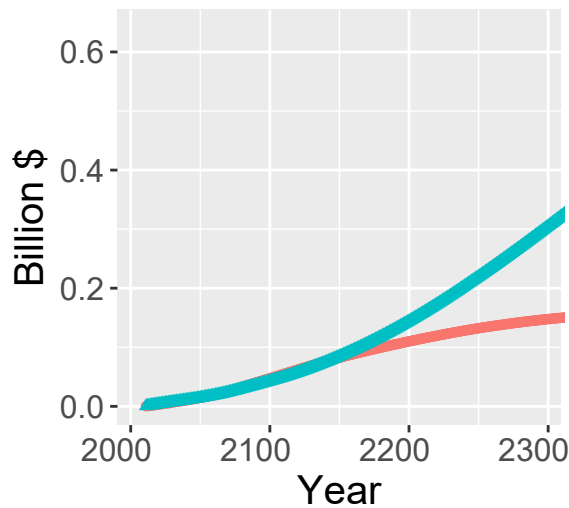

(d) Discounted

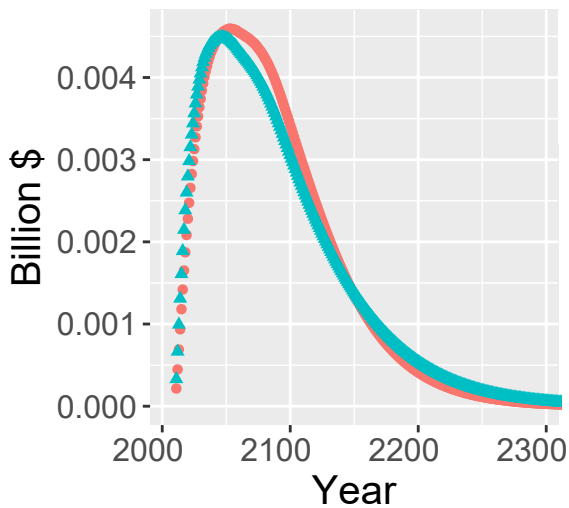

Supplement: SI [file NIHMS1043049-supplement-SI.zip › MetricsPaperCode/results/n2ogridplot.pdf]

(a) Radiative forcing

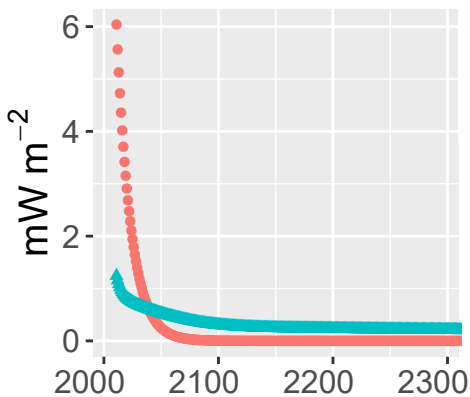

(b) Temperature

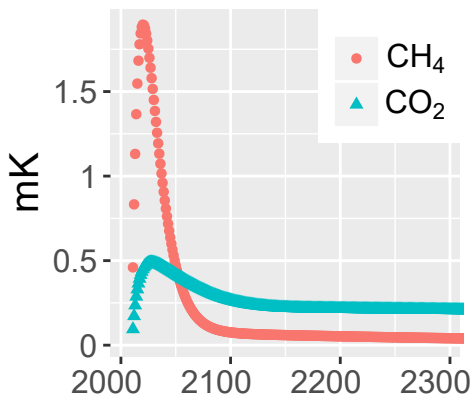

(c) Damages

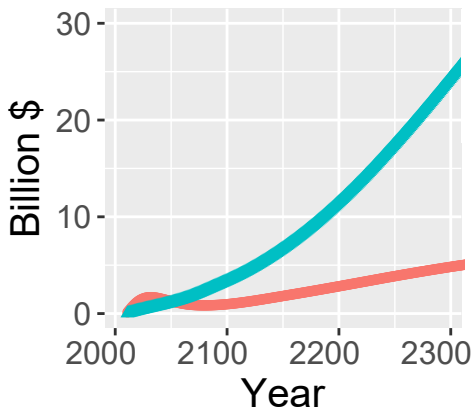

(d) Discounted

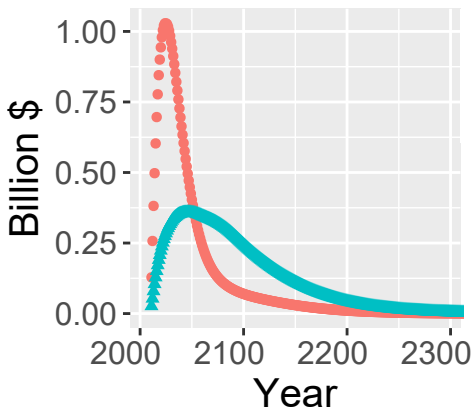

Supplement: SI [file NIHMS1043049-supplement-SI.zip › MetricsPaperCode/results/Figure1.gridplot.pdf]

# GWP and damage ratio

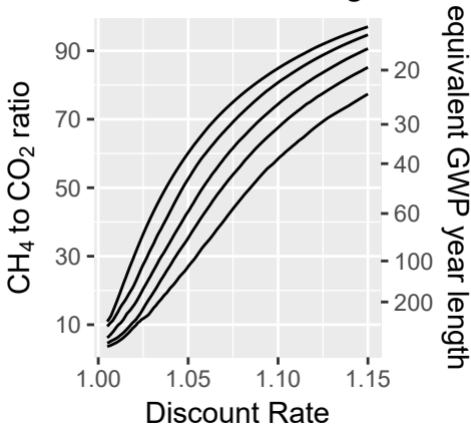

Supplement: SI [file NIHMS1043049-supplement-SI.zip › MetricsPaperCode/results/doubleaxis.pdf]

# CH<sub>4</sub> GWP

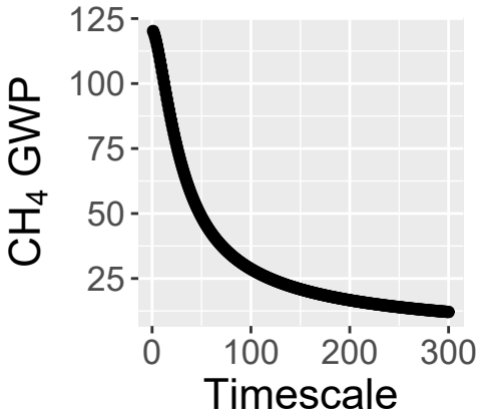

Supplement: SI [file NIHMS1043049-supplement-SI.zip › MetricsPaperCode/results/CH4GWP.pdf]

# CH<sub>4</sub> damage ratio

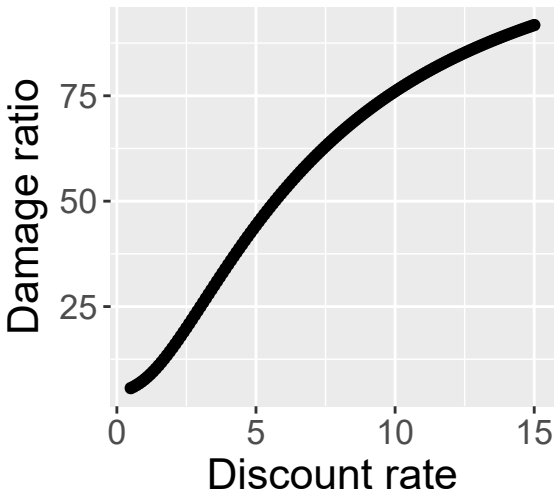

Supplement: SI [file NIHMS1043049-supplement-SI.zip › MetricsPaperCode/results/CH4damageratio.pdf]

equivalent GWP by disc

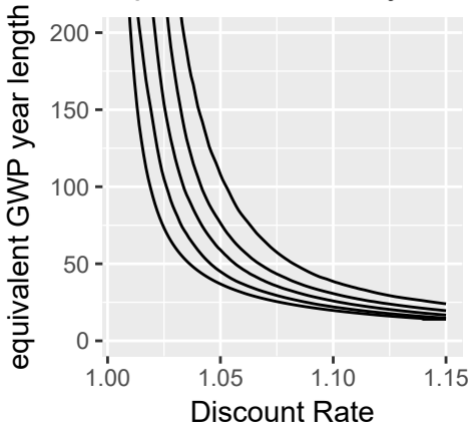

Supplement: SI [file NIHMS1043049-supplement-SI.zip › MetricsPaperCode/results/fig2dummy.pdf]
